# Crystal bending in triple-Laue X-ray interferometry. Part II. Phase-contrast topography

**DOI:** 10.1107/S1600576723002832

**Published:** 2023-05-12

**Authors:** E. Massa, G. Mana, C. P. Sasso

**Affiliations:** a INRIM, Istituto Nazionale di Ricerca Metrologica, Strada delle Cacce 91, 10135 Torino, Italy; b Dipartimento di Fisica, UNITO, Università di Torino, Via Pietro Giuria 1, 10125 Torino, Italy; Oak Ridge National Laboratory, USA; North Carolina State University, USA

**Keywords:** crystal X-ray interferometry, phase-contrast topography, bent crystals, moiré images, thin films, crystal strains

## Abstract

In a previous paper [Sasso *et al.* (2023). *J. Appl. Cryst.* **56**, 707–715], the operation of a triple-Laue X-ray interferometer having the splitting or recombining crystal cylindrically bent was studied. It was predicted that the phase-contrast topography of the interferometer detects the displacement field of the inner crystal surfaces. Therefore, opposite bendings result in the observation of opposite (compressive or tensile) strains. This paper reports on the experimental confirmation of this prediction, where opposite bendings were obtained by copper deposition on one or the other of the crystal sides.

## Abstract 2

The operation model of a triple-Laue interferometer having one of the splitting and recombining crystals bent predicts that the phase-contrast topography detects the displacement field of the inner crystal surfaces. Therefore, it predicts that opposite bendings must result in the observation of opposite strains. This paper reports on the experimental confirmation of this prediction.

## 1. Introduction

Bent silicon crystals have been extensively studied because they are used as optics for the conditioning of X-ray beams and analysers for X-ray spectroscopy (Nesterets & Wilkins, 2008 ▸; Qi *et al.*, 2021 ▸; Kaganer *et al.*, 2020 ▸; Guigay & Sanchez del Rio, 2022 ▸), and to infer the stresses in thin films and devices on substrates (Vaudin *et al.*, 2011 ▸). We are motivated by the search for systematic errors in the measurement of the silicon lattice parameter by crystal X-ray interferometry and the realization of the kilogram by counting silicon atoms (Massa *et al.*, 2011 ▸, 2015 ▸, 2020*a* ▸; Kessler *et al.*, 2017 ▸; Yang *et al.*, 2020 ▸). Therefore, our concern is the phase of the diffracted waves.

Relaxation, reconstruction and oxidation of the surfaces of the splitting and recombining crystals (splitter, mirror and analyser) forming the interferometer cause lattice strains (Quagliotti *et al.*, 2013 ▸). The magnitude of their effect on the lattice parameter measurement was estimated by a finite element analysis, where the surface stress (a fundamental property of the crystal–environment interface) was modelled by an elastic membrane having 1 N m^−1^ tensile strength (Melis *et al.*, 2015 ▸, 2016 ▸; Massa *et al.*, 2020*b* ▸). In addition, a stress difference between surfaces might bend the crystal, and previous studies suggested that the measured lattice spacing might refer to the surface rather than the bulk (Mana *et al.*, 2004*a* ▸,*b* ▸; Apolloni *et al.*, 2008 ▸).

Therefore, in a previous paper (Sasso *et al.*, 2023 ▸), we studied the operation of a triple-Laue X-ray interferometer having one of its splitting and recombining crystals cylindrically bent. Specifically, we noted that the interferometer sees the displacement fields of the splitter’s and analyser’s inner sides. This result and, in turn, the prediction that opposite bending will result in the observation of opposite (compressive or tensile) strains opened the way to an experimental investigation by the phase-contrast imaging of crystal interferometers (Bonse & Hart, 1966 ▸; Bonse *et al.*, 1976 ▸; Ohler *et al.*, 1999 ▸; Bergamin *et al.*, 2000 ▸; Fodchuk & Raransky, 2003 ▸; Massa *et al.*, 2009 ▸, 2020*a* ▸; Drmeyan *et al.*, 2013 ▸, 2017 ▸), which is the subject of this paper.

The paper is organized as follows. Section 2 outlines the operation of the interferometer and the experimental setup for the phase-contrast topography. The measurement procedure, measurement equation and data analysis are described in Section 3. We bent the crystal via the growth of a thin Cu film on one side. The electroless coating is described in Section 3.2. In Section 3.3, the finite element analysis of a coated crystal sets the stage for the measurement design and the understanding of the results. The comparisons between the topography results and the prediction of the interferometer digital twin are given in Section 4. The results confirm the predictions made and deliver further insights into the curvature of a single Si crystal under the effect of stress in a thin film.

## 2. Experimental setup

**Figure 1:**
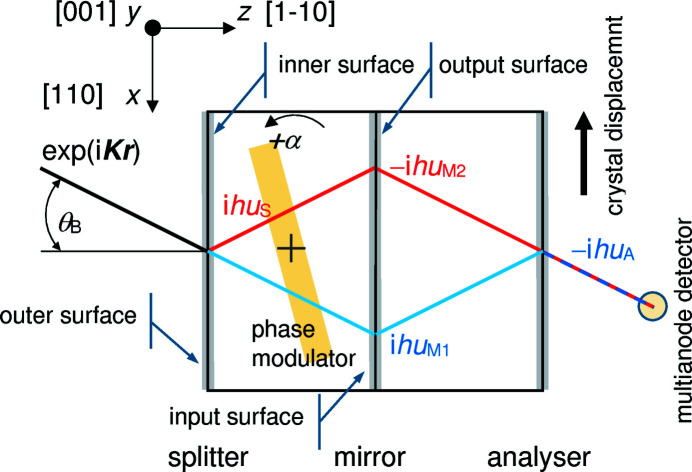
X-ray phase-contrast topography. The X-ray paths are drawn in red (RRT path) and blue (TRR path). The Bragg angle is out of scale. The phase delay of each reflection is given. The X-ray crossings with the mirror are spaced by 4 mm. Adapted from Massa *et al.* (2020*a* ▸).

Fig. 1 ▸ shows the experimental setup (Hart & Bonse, 1970 ▸). A first crystal (splitter) splits 17 keV X-rays from a fixed-anode (0.1 × 10) mm^2^ Mo *K*α source, and the rays are recombined, via two mirror-like crystals, by the last crystal (analyser). The X-ray interference is extremely sensitive to any local mismatch of the crystal lattices in a direction orthogonal to the diffracting (220) planes. A displacement in any interferometer crystal equal to one plane creates a 2π phase shift. Therefore, the interference result is a moiré pattern encoding the differences between the displacement fields of the four crystals (Chetwynd *et al.*, 1998 ▸; Lang, 2006 ▸).

X-rays are collimated by a (0.5 × 16) mm^2^ slit placed in front of the interferometer. The interference fringes are imaged onto a multianode photomultiplier tube through a vertical pile of eight 1 mm NaI(Tl) scintillators, spaced by 1 mm shades.

The splitter, mirror and analyser are (35 × 18 × 0.8) mm^3^, spaced 10.2 mm apart, and protrude from a common base. Since the X-ray source and detector are 0.8 and 0.3 m, respectively, from the mirror, the images of the scintillator pixels projected on the mirror are, on average, (1 × 3) mm^2^. The projected image of the scintillator pile is 13 mm in height.

As shown in Fig. 1 ▸, we imaged the moiré pattern by shifting the interferometer in 0.5 mm steps along the *x* axis and detecting the interference fringes in 61 adjacent (1 × 13) mm^2^ vertical (overlapping) slabs subdivided into 8 (overlapping) pixels of (1 × 3) mm^2^. Therefore, the (61 × 8) pixels image a (30 × 10) mm^2^ area on the interferometer crystals, by using the coordinates of the pixel centres.

## 3. Measurement procedure

### 3.1. Data analysis

A review of phase-contrast X-ray imaging based on crystal interferometry is given by Momose (2002 ▸). In a geometric optics model of the interferometer (with the positive exponent choice representing a plane wave with positive wavenumber *K*, see Fig. 1 ▸), each crystal delays the phase of the reflected X-rays (relative to the forward transmitted X-rays) by 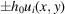 , where 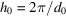 is the perfect-crystal reciprocal vector, 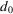 is the perfect-crystal spacing of the diffracting planes, and 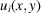 (*i* = A, M1, M2, S) is the *x* component of the displacement field of the bent splitter (S), mirror (M1, M2) or analyser (A). The sign is positive if 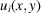 is in the same direction as the *x* component of the incident-beam wavevector and negative otherwise.

The difference between the phase delays, 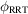 and 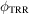 , along the two paths reaching the observation plane – one performing two reflections (R) followed by one transmission (T), the other one transmission followed by two reflections – is 

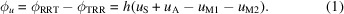

According to the dynamical theory model of a triple-Laue interferometer (Sasso *et al.*, 2023 ▸), the displacement fields 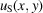 and 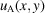 refer to the (splitter and analyser) inner sides (see Fig. 1 ▸) and 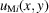 are the means of the displacement fields of the two mirror surfaces (see Fig. 1 ▸).

A plastic sheet, 1 mm thick, is placed between the splitter and mirror. With the positive exponent choice representing a plane wave (see Fig. 1 ▸), it modulates the interference phase by 

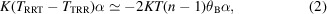

where α is the angle of rotation (positive if counterclockwise), *T* the thickness, 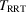 and 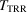 the lengths of the X-ray paths, 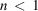 the index of refraction, and 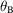 the Bragg angle. The linearization is valid if 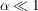 rad.

The interference fringes are detected by each of the eight photomultiplier channels. The measurement equation is 

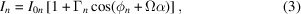

where 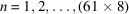 label the image pixel, 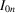 is the average count rate, 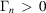 the contrast, and 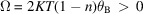 the period.

The phases 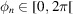 in the 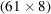 image pixels are recovered by least-squares estimations, with the constraints 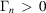 and 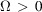 . After the unwrapping, we used 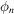 to infer the displacement field 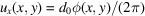 .

Since we are interested only in the strain change after coating, a reference phase survey without any coating is taken in advance and subsequently subtracted to isolate the dis­place­ments induced by the Cu film. Since positive phase gradients correspond to displacements of the splitter and analyser lattices in the *x* direction and the opposite for the mirror lattice, tensile and compressive strains can be distinguished. Also, since 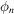 is recovered modulo 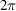 , a constant displacement is undetectable.

In (1), we neglected minor contributions coming from the phase of the crystals’ reflection and transmission coefficients; this phase is sensitive to deviations of the crystal surfaces from being plane and parallel and to the misalignment and spacing of the diffracting planes. These contributions are discussed by Mana & Vittone (1997 ▸), Bergamin *et al.* (2000 ▸) and Sasso *et al.* (2023 ▸) and amount to a few per cent of a period. Also, since we subtracted the phase map of the naked interferometer, the interferometer and phase modulator geometry and intrinsic strains are irrelevant.

### 3.2. Cu coating

The coating of the crystals was carried out by electroless galvanic deposition in a water solution of copper(II) nitrate, Cu(NO_3_)_2_ (60 g l^−1^), and ammonium fluoride, NH_4_F (30 g l^−1^). The copper plates the silicon surface and, simultaneously, the oxidized silicon is removed by HF^−^ to form water-soluble silicates and a clean interface between the Cu layer and the silicon crystal surface. The overall stoichiometric reaction is (Mendel & Kuei-Hsuing Yang, 1969 ▸) 

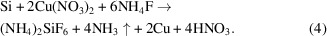

The growth of the Cu film and the generated stress depend on the solution composition and temperature (20°C). Therefore, based on the results given by Massa *et al.* (2020*a* ▸), we coated both crystal sides to estimate the stress from a preliminary phase-contrast image of the induced strain. We removed the coating from one of the surfaces only after the surface stress was estimated in this way.

### 3.3. Finite element analysis

We set up a finite element analysis of the coated interferometer crystals, modelled as a 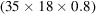 mm^3^ Si crystal (IT Center for Science, 2020 ▸). Since the intrinsic displacement field of the naked interferometer was subtracted from the coating-induced one, gravity was switched off and the self-weight displacements (already detected before the coating) were not included in the analysis.

**Figure 2:**
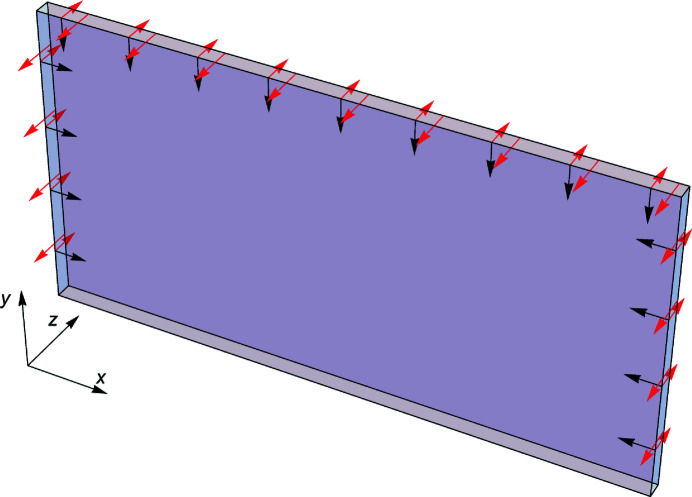
Surface stress modelling by forces per unit length applied orthogonally to edges and lying in the crystal surfaces. Black: forces acting on the coated *z* = 0 mm surface. Red: forces acting on the crystal rim. The boundary conditions specify a displacement field equal to zero at the bottom, *y* = 0 mm, surface.

**Figure 3:**
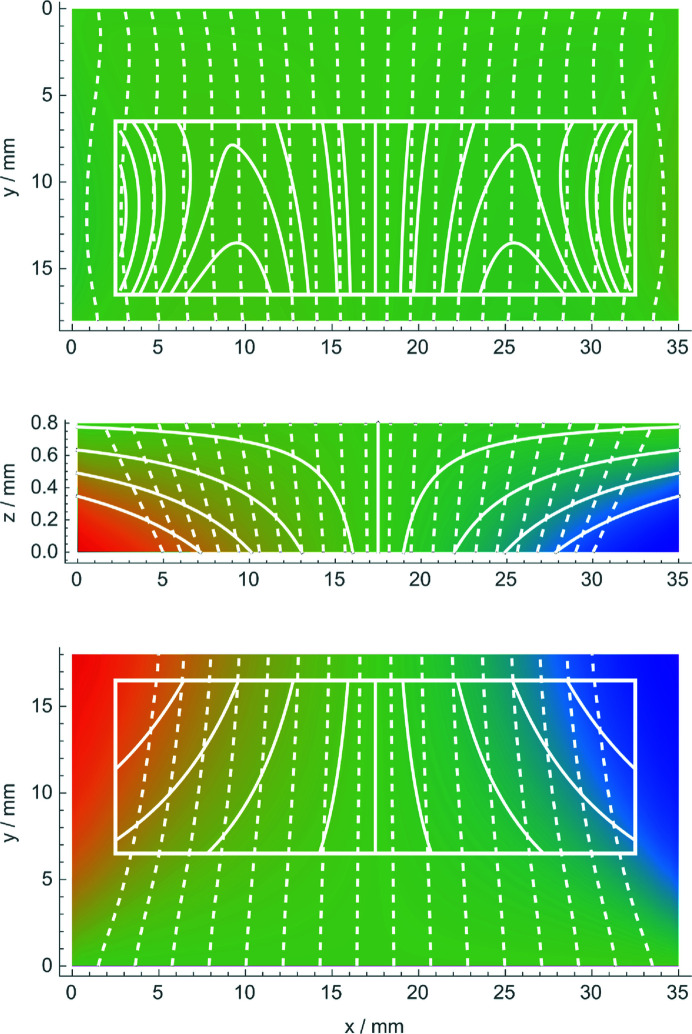
Finite element analysis of a bent crystal: displacement field 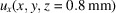 (top), 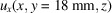 (middle) and 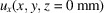 (bottom). The *z* = 0 mm surface is coated, and the surface stress is 6 N m^−1^. The colour scale is from −1.4 nm (blue) to 1.4 nm (red). White lines are contours of constant displacement (solid) and the diffracting planes with displacements magnified (dashed). The rectangle indicates the imaged area.

As shown in Fig. 2 ▸, the effect of the Cu film was simulated by an equiaxial and uniform compressive surface stress, τ, modelled as forces per unit length applied orthogonally to relevant edges and lying in the crystal surfaces. We set Dirichlet boundary conditions on the bottom surface, *y* = 0 mm, by specifying a displacement field equal to zero, and used an anisotropic stiffness matrix (Quagliotti *et al.*, 2013 ▸; Zhang *et al.*, 2014 ▸). The *x* and *z* axes of the finite element model are parallel to the crystallographic directions (110) and 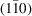 , the *y* axis points upwards, and the reference frame origin is the crystal’s bottom-left corner. A typical result is shown in Fig. 3 ▸. In contrast to our naive expectation, Fig. 3 ▸ shows that the coating does not induce tensile stress on the opposite (naked) surface, which is almost unstrained.

To simplify the fit to the experimental data of the *z* = 0 mm and *z* = 0.8 mm sections of the *x* displacements obtained via the finite element analysis [in the top, central, (30 × 10) mm^2^ imaged part, see the white rectangle in Fig. 3 ▸], we used a polynomial that was as simple as possible. In Fig. 3 ▸, the contour lines are obtained by fitting this polynomial to the finite element analysis. The residual standard deviations are a few per cent of the maximum displacement.

The approximating polynomial is (see Appendix *A* [App appa]) 

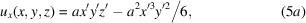

where 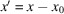 , 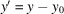 , 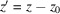 , 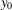 is a model parameter without a specific physical meaning, 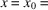 17.5 mm is the symmetry plane and 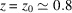 mm is the neutral plane. The stressed (coated) surface is the *z* = 0 mm one, 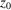 is equal to the crystal thickness, and the neutral plane is the uncoated surface. The last term in (5*a* [Disp-formula fd5a]) encodes the *x* component of the (geometrical) radial displacement of the bent crystal and is relevant only if 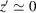 (see Appendix *A* [App appa]).

The polynomial (5*a* [Disp-formula fd5a]) describes the effects of two bendings. The first bending occurs about a vertical axis in the 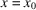 plane and has curvature 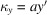 . It takes the differential stress of the front and rear surfaces into account. The vertical increase of the curvature encodes the consequence of the zero displacements on the bottom surface. The *y* = const. sections of (5*a* [Disp-formula fd5a]) were assumed in solving the Takagi–Taupin equations for the dynamical X-ray propagation (Sasso *et al.*, 2023 ▸).

The second bending occurs about an axis parallel to 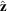 in the same 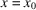 plane and has curvature 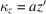 . It encodes the vertical increase of the (compressive) strain, which is zero at the bottom and maximum at the top. The curvature depends on *z* because the neutral plane 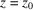 is (almost) unstrained.

When both the front and rear surfaces are coated and equally stressed, the bending in the horizontal plane disappears. This means that 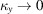 . In this case, the polynomial approximation simplifying the fit of the finite element analysis to the experimental data is given by the limit of (5*a* [Disp-formula fd5a]) as 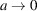 and 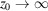 with 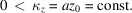 , which is 

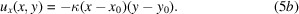




The experimentally determined *x* and *y* axes might be slightly rotated with respect to the crystallographic directions (110) and (001), they might deviate from being perfectly orthogonal, and their origin might be displaced. Furthermore, the assumption of uniform surface stress might not be valid.

Therefore, to accommodate these degrees of freedom, the actual polynomial used to fit the finite element analysis to a stressed phase-contrast image, 

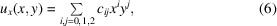

is obtained from (5*a* [Disp-formula fd5a]) or (5*b* [Disp-formula fd5b]), where we allowed for rotations and translations of the *x* and *y* axes and omitted the 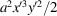 term because it is irrelevant. The polynomial fitting the analysis to the neutral plane image, 

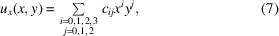

is similarly obtained from the last term of (5*a* [Disp-formula fd5a]).

We used the finite element analysis for two complementary purposes. The first is predicting the displacements on the surfaces of the splitter, mirror and analyser, given the surface stress due to the Cu coating on one of the crystal surfaces. We note that the analysis linearity allowed us to scale the displacements linearly with the surface stress.

The second purpose is to compare predictions and observations by fitting the analysis to the phase-contrast images. The comparison was carried out via the Gauss curvature 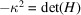 (Weisstein, 2023 ▸), where *H* is the Hessian of the polynomial (6) best fitting the data. The reason for this choice is the invariance of the Gauss curvature under the distance-preserving transformations of (5*a* [Disp-formula fd5a]) and (5*b* [Disp-formula fd5b]) into (6). In addition, we used the mean strain at the top of the imaged area.

## 4. Phase-contrast topography

In the next sections, we will discuss the cases when the bent crystal is the mirror, the splitter or the analyser. As shown in Appendix *B* [App appb], opposite bendings of the same crystal were achieved by flipping the interferometer by 180°. In all cases, to infer the surface stress τ induced by the Cu coating, firstly, we coated both sides of the crystal and fitted the polynomial (6) to the observed displacements (Massa *et al.*, 2020*a* ▸). Next, assuming the same surface stress, we used the best-fit value of τ to predict, via the finite element analysis and the dynamical theory model of the interferometer operation, the displacement fields that will be detected by the (front and rear) topography carried out after the coating was removed from one side by FeCl_3_ etching.

We verified that the finite element analysis is insensitive to changes in the crystal thickness (assumed not to exceed 50 µm) and residual surface stress of the naked surface (assumed not to exceed 0.5 N m^−1^).

As regards the effects of the (vertical and horizontal) gradients of the tilt, 

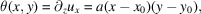

and spacing, 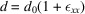 , where 

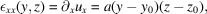

of the diffracting planes on the phase-contrast images, the following considerations hold.

With the maximum τ = 6 N m^−1^ surface stress generated by the Cu coating, the horizontal and vertical gradients of θ are less than 1 and 0.5 µrad cm^−1^, respectively, θ being equal to zero at 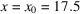 mm [see Fig. 3 ▸ (middle)]. From this viewpoint, the phase shift between different points of the phase-contrast image due to the tilt of the diffracting planes is the same as what would be observed by rotating the crystal by the same θ angle. It never exceeds a few per cent of a period (Mana & Vittone, 1997 ▸).

The change in the diffracting-plane spacing affects the interference phase in two ways. Firstly, it mimics a crystal rotation, though in the opposite direction for the forward-transmitted and diffracted beams. Since, with τ = 6 N m^−1^, the maximum apparent rotation is less than 12 nrad, its contribution to the phase can be safely neglected.

Secondly, when one of the interferometer crystals is bent, the rays interfering parallelly leave the source from different points and propagate in different directions (Sasso *et al.*, 2023 ▸). In turn, these differences cause optical path differences and raise questions about source coherence. Investigations would require extending the two-dimensional interferometer model given by Sasso *et al.* (2023 ▸) to three dimensions and partially coherent illumination (Sasso *et al.*, 2022 ▸). Though we do not have a full understanding of the relevant physics, the phase-contrast images did not provide clues about problems related to them.

### 4.1. Mirror

**Figure 4:**
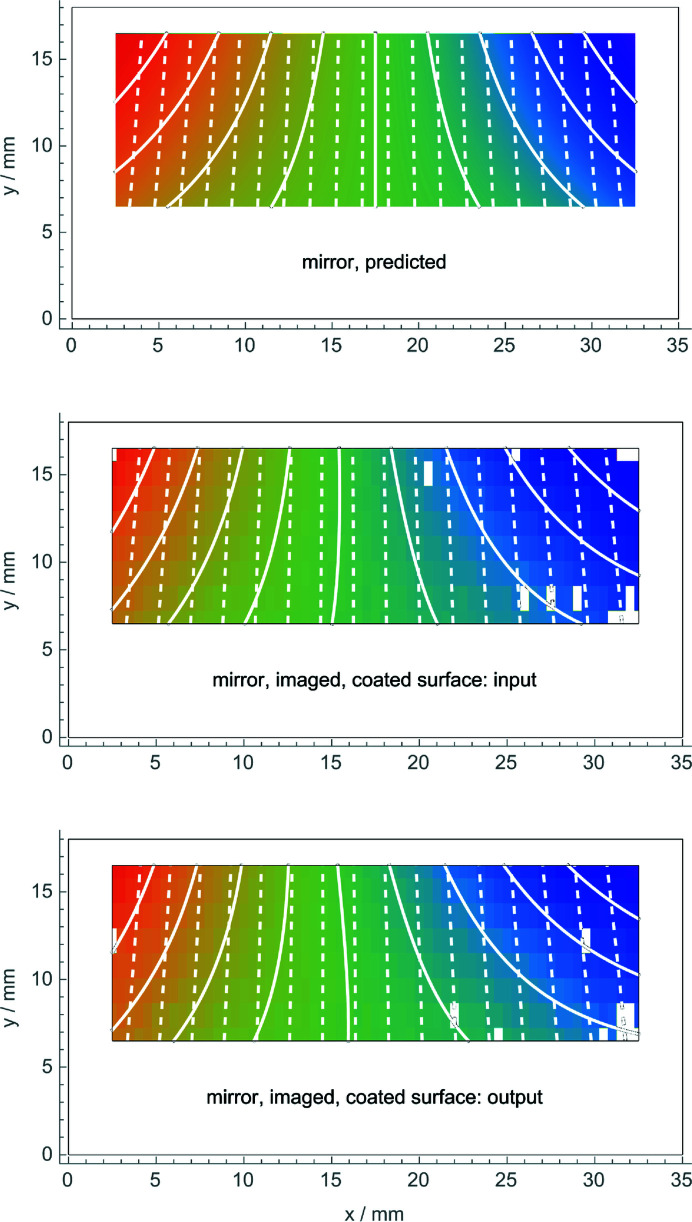
Top: mean displacement field, see (8), predicted when only one side of the mirror is coated. Middle and bottom: observed displacements. The colour scale is from −89 pm (blue) to 89 pm (red). White lines are contours of constant displacement (solid) and the diffracting planes with displacements magnified (dashed). The white pixels indicate outliers excluded from the analysis.

When only one mirror side is coated, no matter if it is the input or the output one (see Fig. 1 ▸), the dynamical theory predicts that the phase-contrast topography will image the average of the displacements on the input, *z* = 0 mm, and output, *z* = 0.8 mm, surfaces. The surface stress, τ = 1.1 (1) N m^−1^, was estimated from the displacements observed when both sides of the mirror are coated. This value was used in the finite element analysis to calculate the mean displacement field when only one side is stressed, 

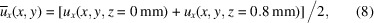

which is shown in Fig. 4 ▸ (top).

The mean top-strain and curvature, −5.8 (2) pm mm^−1^ and 0.29 (2) × 10^−6^ m^−1^, given in Table 1 ▸ are the averages of the values obtained when the crystal thickness, stress gradients of the coated side, residual stress of the naked side and surface stress of the crystal rim were varied to take into account the limited knowledge of their values. The relevant uncertainties are given in parentheses.

After removing the coating from one side, we imaged again the mirror displacements: firstly, with the interferometer placed in such a way that the input surface was the coated one and, secondly, with the interferometer turned by 180° so that the output surface was the coated one. An example of the measurement procedure is given in Appendix *B* [App appb]. The results are shown in Fig. 4 ▸ (middle and bottom), where the contours of constant displacement are calculated from the polynomial (6) best fitting the data, and Table 1 ▸. The experimentally determined values of the mean top-strain and curvature in the two cases are nearly identical, agree with the predicted ones, and confirm that the observed displacements do not detect the bending.

### 4.2. Splitter and analyser

When the bent crystal is the splitter or the analyser, the dynamical theory predicts that the observed displacements refer to the surface inside the interferometer (see Fig. 1 ▸). To test this prediction, we reset the interferometer by FeCl_3_ etching and coated both sides of one of the extremal crystals – which will be the splitter or analyser depending on its mounting towards the source or detector. Since the sensitivity of the fringe phase to the splitter and analyser displacements is half that of the mirror and, also, since we expected to image no displacements when their coated surface is external, we increased the Cu thickness to generate the maximum detectable displacement, which is set by the minimum pitch of the moiré fringes still observable, a few millimetres.

After imaging the displacements due to the two-side coating, we estimated the newly induced surface tension, removed the coating from the outer side, and imaged the displacements again. The coated crystal operated, firstly, as the splitter and, subsequently, as the analyser. In both cases, the coated (stressed) surface was internal. Eventually, the interferometer was reset, and the procedure was repeated, but now removing the coating from the inner side so that the coated (stressed) surface was external. The measurement sequence is illustrated pictorially in Appendix *B* [App appb].

**Figure 5:**
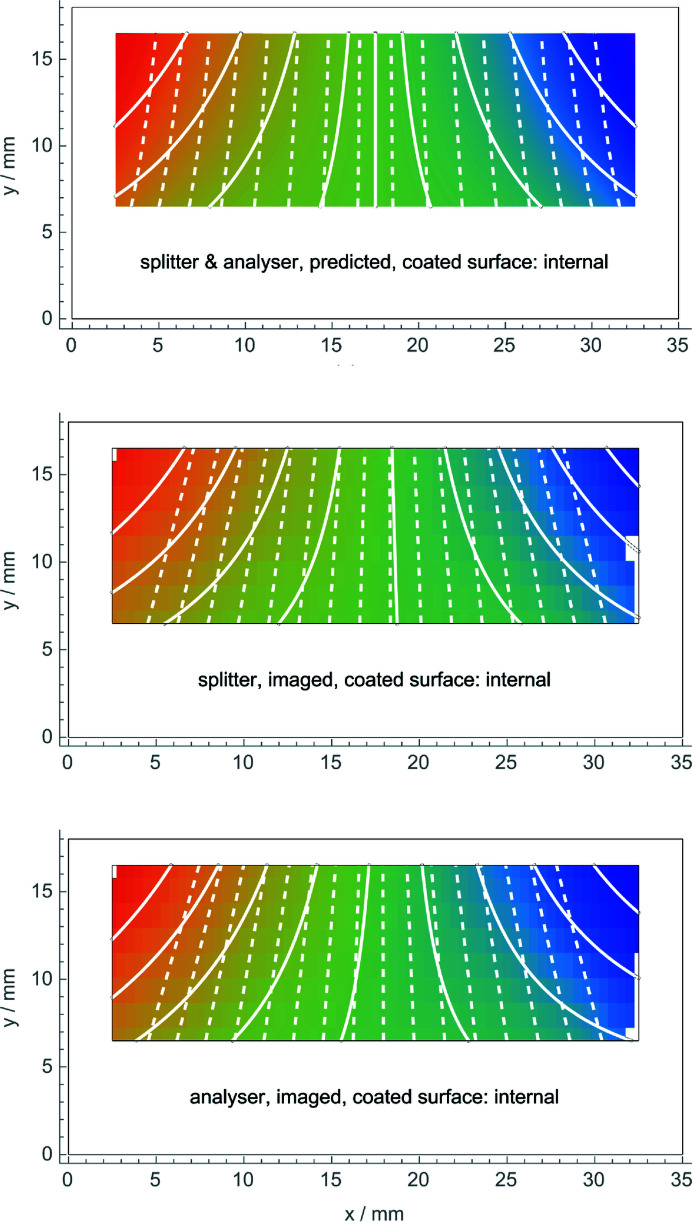
Top: predicted observation of the displacement field when the inner side of the splitter or the analyser is coated. Middle and bottom: observed displacements. The colour scale is from −1 nm (blue) to 1 nm (red). White lines are contours of constant displacement (solid) and the diffracting planes with displacements magnified (dashed). The white pixels indicate outliers excluded from the analysis.

**Figure 6:**
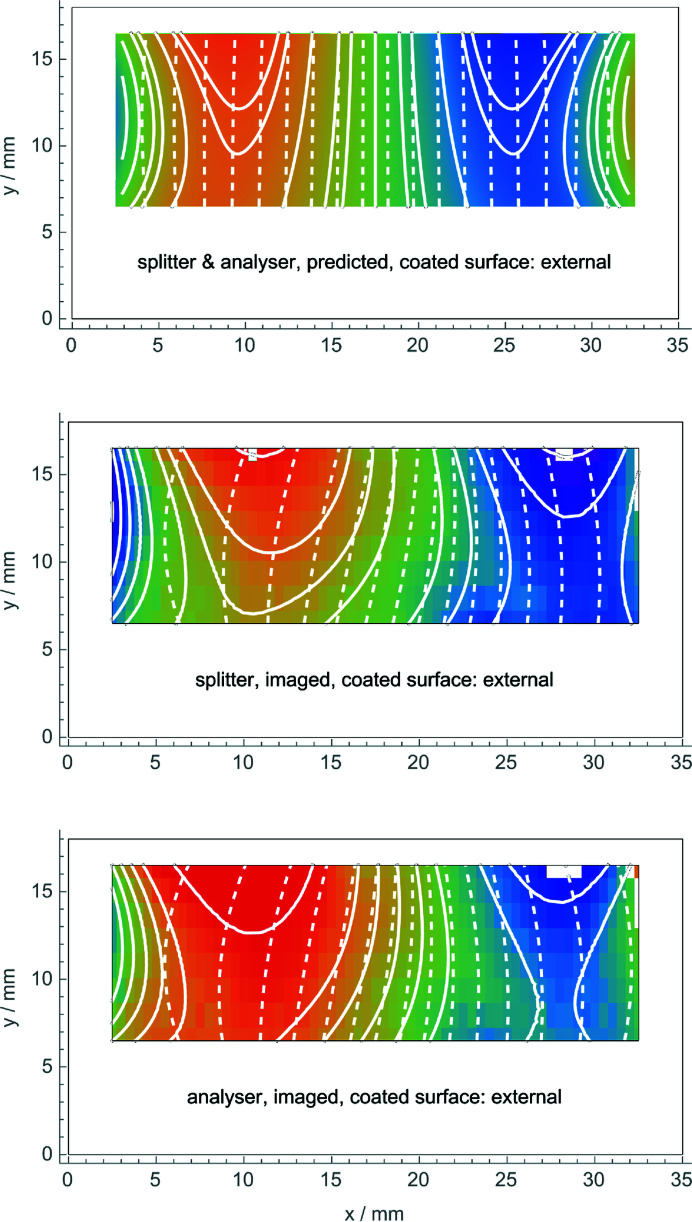
Top: predicted observation of the displacement field when the outer side of the splitter or the analyser is coated. Middle and bottom: observed displacements. The colour scale is from −110 pm (blue) to 110 pm (red). White lines are contours of constant displacement (solid) and the diffracting planes with displacements magnified (dashed). The white pixels indicate outliers excluded from the analysis.

Figs. 5 ▸ and 6 ▸ (top) show the predicted displacements. The surface stress used in the finite element analysis was τ = 6.1 (6) N m^−1^ in the first coating, Fig. 5 ▸ (top), and τ = 6.3 (6) in the second one, Fig. 6 ▸ (top).

Fig. 5 ▸ (middle and bottom) shows the observed displacements when the coated (stressed) surface of the splitter (analyser) was internal. We note that the contours of constant displacement were calculated from the polynomial (6) best fitting the data. To take these images, the interferometer was mounted, firstly, in such a way that the coated crystal operated as the splitter and, secondly, with the interferometer turned by 180° so that the coated crystal operated as the analyser.

Table 1 ▸ compares the predicted and observed curvatures, κ, and mean stresses at the top of the imaged area, 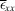 . The uncertainties (in parentheses) associated with the observations were roughly estimated by comparing different surveys. Those associated with the predictions take into account the uncertainties of the crystal thickness, stress gradients of the coated side, residual stress of the naked side and surface stress of the crystal rim.

The estimated value of κ ( 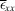 ) is lower (higher) than that fitting the observed displacements. However, the predicted and the observed ratios of the κ value associated with the one- and two-side coating cases agree quite well. The same is true for the ratios of the 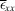 values.

**Figure 7:**
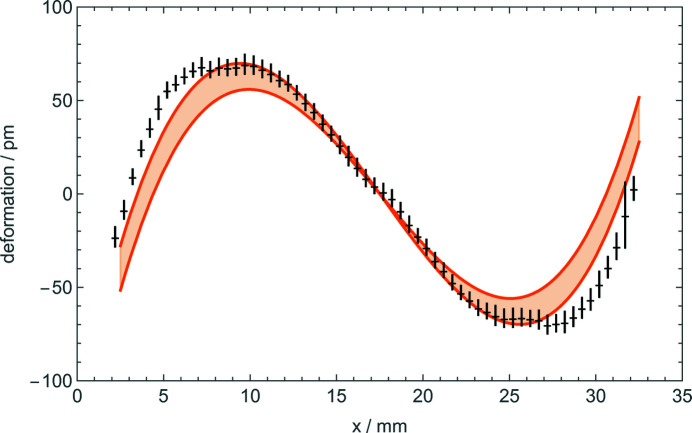
Predicted (orange lines) and observed (black crosses) displacement fields of the splitter and analyser when their outer side is coated. The displacements are averaged over the 10 mm height of the imaged area. Since they are expected to be identical, the observed (splitter and analyser) displacements were averaged. The bars indicate the uncertainties, set equal to two standard deviations. The orange lines make reference to the surface stress applied or not applied to the crystal rim.

Fig. 6 ▸ (middle and bottom) shows the displacements observed when the coated (stressed) surface of the splitter (analyser) was external. In this case, the contours of constant displacement were calculated from the polynomial (7) best fitting the data. The interferometer was again mounted, firstly, with the coated crystal operating as the splitter and, secondly, as the analyser. As predicted, the displacements are almost identical and almost null. The agreement between predictions and observations is confirmed by Fig. 7 ▸, where they have been averaged over the 10 mm height of the imaged area.

**Figure 8:**
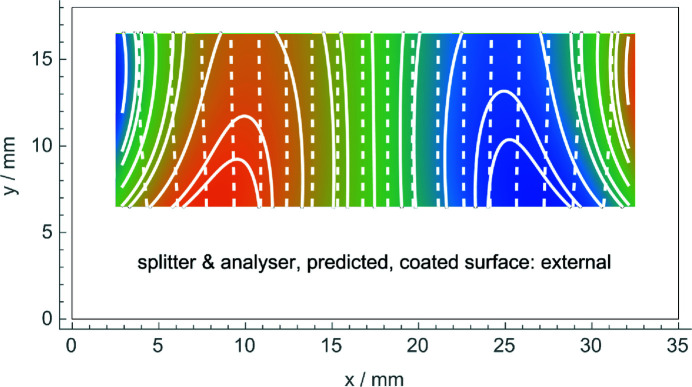
Predicted observation of the displacement field when the outer side of the splitter or the analyser is coated and the elastic anisotropy of silicon is neglected. The colour scale is from −83 pm (blue) to 83 pm (red). Comparison with Fig. 6 ▸ shows that, neglecting the anisotropy, we fail to predict correctly the result of the phase-contrast topography.

As shown in Fig. 8 ▸, when neglecting the elastic anisotropy of silicon, the finite element analysis fails to predict correctly the result of the phase-contrast topography. To our knowledge, this is the first observation of the elastic anisotropy at the atomic scale and shows the extreme sensitivity of X-ray interferometry to the strains of the splitting and recombining crystals.

## 5. Conclusions

We used phase-contrast imaging to test the dynamical theory predictions of the sensitivity of a triple-Laue crystal interferometer to the bending of the splitting and recombining crystals. Specifically, we checked the prediction that the interferometer is insensitive to the concave or convex bending of the mirror (meaning that it senses the mean displacements) but sensitive to the splitter and analyser bending (meaning that it senses the displacements on their inner surface).

In particular, we compared the displacement fields predicted via the finite element analysis against those observed via phase-contrast images. Opposite bendings were induced by a Cu film plated on one or the other side of the crystal. The detection of strains as small as 1 nm m^−1^ proved possible.

The results confirm the theoretical predictions. As shown in Fig. 4 ▸ (middle and bottom), the same mirror displacements were observed, no matter whether the bending was towards the source or the detector. Also, the two surveys agree with the predicted mean (see Fig. 4 ▸, top) of the front and rear displacement fields.

Comparing Figs. 5 ▸ and 6 ▸ (middle and bottom), we see that different displacements were observed, depending on whether the splitter or analyser bending was towards the inner or outer side of the interferometer. As shown in the figures, the observed displacements do not depend on the bent crystal working as the splitter or the analyser. Also, in this case, observations and predictions agree [see Figs. 5 ▸, 6 ▸ and 7 ▸ (top)].

The qualitative and quantitative differences observed depending on the stressed surface of the analyser being external or internal to the interferometer might impact the absolute measurement of the silicon lattice parameter and the kilogram realization by counting silicon atoms. Future work will investigate experimentally the surface stress induced by crystal oxidation.

We observed the anisotropic elastic behaviour of silicon at the atomic scale. Anisotropy determines a residual strain pattern of the (inner) neutral surface of the splitter or the analyser (which is opposite to the Cu-coated one) that is qualitatively different from that predicted by an isotropic model (compare Figs. 6 ▸ and 8 ▸). Strictly speaking, the complex displacement pattern observed and shown in Fig. 6 ▸ cannot be predicted by the (two-dimensional) interferometer model that prompted this work. In fact, it assumed a constant strain on the crystal surfaces. Therefore, the agreement shown in Fig. 6 ▸ between the expected and observed displacements is a clue to the more general validity of the predictions made.

Our results are also a successful test of the dynamical theory of X-ray diffraction in deformed crystals, where, instead of the propagated intensity, we considered the phase changes in the reflection and transmission of the X-rays.

From a practical viewpoint, X-ray interferometry allows the investigation of stress in thin films in a new way. The phase-contrast topography may provide the basis for new insights into the relationship between the silicon substrate and thin films, and their application to device design and manufacturing.

## Figures and Tables

**Figure 9:**
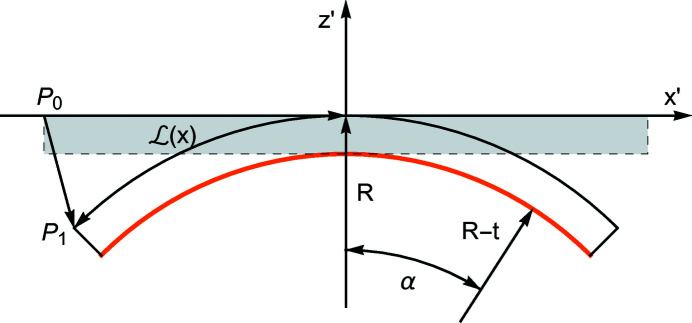
Geometry of the bent crystal; 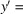 const. section. The axis *x* is normal to the diffracting planes, and the axis *z* is normal to the crystal surfaces. The orange line indicates the Cu coating. The rear surface lies in the neutral plane, 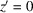 .

**Figure 10:**
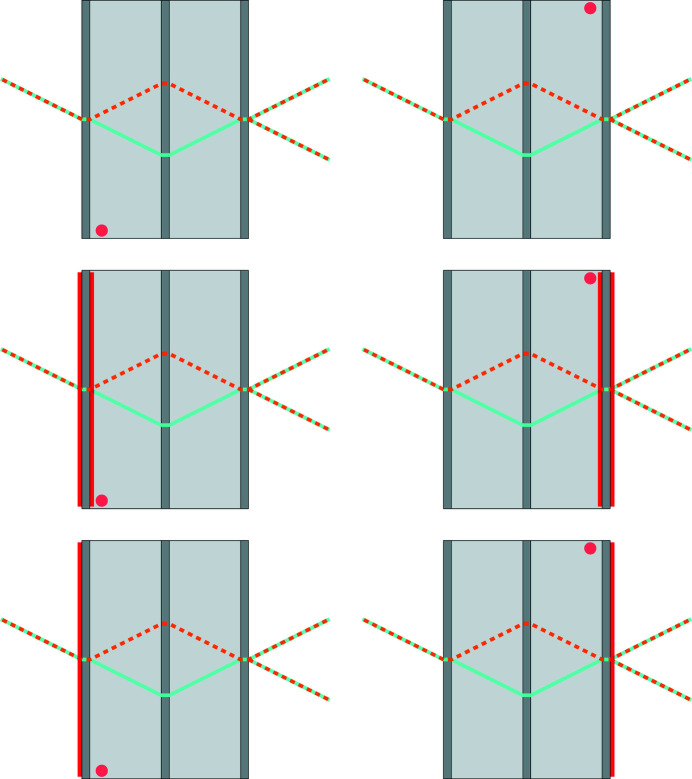
Example of the measurement sequence. Top: front and rear topographic surveys are carried out to infer the differential displacements. Middle: front and rear surveys are carried out to determine the surface stress induced by the Cu coating. Bottom: front and rear surveys are carried out to observe the displacements yielded by the Cu coating on the external surface of the splitter and analyser. The red lines indicate the coated surfaces. The orange marker indicates the interferometer front and rear orientations.

**Table 1:** Comparison of the predicted and observed displacements The surface stresses τ have been estimated by fitting the polynomial (6) to the observed displacements when both sides of the crystal are coated. When only one side is coated, the predictions assumed the same surface stress value. κ and 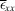 are the curvature and the mean surface stress at the top, respectively, of the polynomial (6) best fitting the predicted and observed displacements (see Figs. 4 ▸, 5 ▸ and 6 ▸). σ is the fractional standard deviation (to the maximum displacement) of the residuals of the polynomials (6) and (7) best fitting the predicted and observed displacements. When the coated side of the splitter and analyser is external (last two lines) κ and 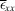 are meaningless.

			Prediction			Observation		
Bent crystal	Coated surface	τ (N m^−1^)	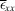 (pm mm^−1^)	κ (10^−6^ m^−1^)	σ (%)	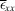 (pm mm^−1^)	κ (10^−6^ m^−1^)	σ (%)
Mirror	Both	1.1	−12	0.56	1.5	−11 (1)	0.59 (6)	1.4
Mirror	Input	1.1	−5.8 (2)	0.28 (2)	1.5	−5.5 (5)	0.28 (3)	2.1
Mirror	Output	1.1	−5.8 (2)	0.28 (2)	1.5	−5.4 (5)	0.30 (3)	2.0
								
Splitter/analyser	Both	6.1	−65	3.1	1.5	−58 (6)	3.4 (3)	2.4
Splitter	Internal	6.1	−63 (2)	3.2 (2)	4.8	−57 (6)	3.2 (3)	3.1
Analyser	Internal	6.1	−63 (2)	3.2 (2)	4.8	−62 (6)	3.5 (3)	3.0
								
Splitter/analyser	Both	6.3	−67	3.2	1.5	−65 (6)	3.3 (3)	3.5
Splitter	External	6.3	–	–	2.3	–	–	6.9
Analyser	External	6.3	–	–	2.3	–	–	6.9

## Appendix A Bending model

With reference to the geometrical model of the cylindrically bent crystal shown in Fig. 9 ▸, in the  const. sections, we describe the neutral plane by the parabola  . The arc length of its portion from zero to  is

where  is the curvature,  and *t* is the crystal thickness. Hence, the displaced point  of the neutral surface was initially in  (see Fig. 9 ▸). In general, the initial point  , lying in a generic  const. surface, is displaced to   , where  is the neutral plane,  is the additional displacement of the points outside the neutral plane and  .

The *x* component of the displacement vector from the initial to the final points is

where we assumed that the curvature  depends linearly on the vertical coordinate  . We note that, in our case, the neutral plane coincides with the rear,  , surface.

## Appendix B Measurement procedure

As an example, Fig. 10 ▸ shows the measurement procedure when the bent crystal is the splitter or the analyser, and the crystal concavity is towards the outside of the interferometer. The sequence of the measurements is the same, *mutatis mutandis*, in the other cases considered.

Firstly, after removing the previous coatings, two (front and rear) topographic surveys are carried out to acquire the (zero) differential displacements  .

Next, one of the extremal crystals (splitter or analyser, depending on its mounting towards the source or detector) is coated on both sides. Two (front and rear) surveys are carried out to infer the induced surface stress after subtracting the relevant zero displacements. This subtraction allows us to observe the coating effect unambiguously.

Eventually, the Cu coating is removed from the inner surface, and the last two (front and rear) surveys are carried out. The zero displacements, observed with the interferometer having the same orientation, are again subtracted from the observed displacements.
